# Proteomic Analysis of Serum and Urine of HIV-Monoinfected and HIV/HCV-Coinfected Patients Undergoing Long Term Treatment with Nevirapine

**DOI:** 10.1155/2014/315824

**Published:** 2014-12-17

**Authors:** Jeerang Wongtrakul, Thananya Thongtan, Sittiruk Roytrakul, Benjawan Kumrapich, Kanokwan Janphen, Jutarat Praparattanapan, Khuanchai Supparatpinyo, Duncan R. Smith

**Affiliations:** ^1^Research Institute for Health Sciences, Chiang Mai University, Chiang Mai 50200, Thailand; ^2^Department of Biochemistry, Faculty of Medicine, Chulalongkorn University, Bangkok 10330, Thailand; ^3^Proteomics Research Laboratory, Genome Institute, National Center for Genetic Engineering and Biotechnology, Pathum Thani 12120, Thailand; ^4^Department of Medicine, Faculty of Medicine, Chiang Mai University, Chiang Mai 50200, Thailand; ^5^Institute of Molecular Biosciences, Mahidol University, Salaya Campus, 25/25 Phuttamonthol Sai 4, Nakorn Pathom 73170, Thailand

## Abstract

Nevirapine (NVP) is an effective nonnucleoside reverse transcriptase inhibitor (NNRTI) of particular interest as it is often used in resource limited countries. However, one of the main concerns with the use of NVP is hepatotoxicity and elevation of liver enzymes as a consequence of highly active antiretroviral therapy (HAART) containing NVP is more often reported in HIV patients coinfected with hepatitis C virus than in HIV-monoinfected patients. To discover possible markers of NVP induced hepatotoxicity, serum and urine samples from twenty-five HIV or HIV/HCV patients, all of whom had received NVP continuously for at least four months, and healthy controls were subjected to in-solution or in-gel proteomic analysis. A total of 83 differentially regulated proteins consisted of 34 proteins identified in serum by in-solution analysis, 2 proteins identified from serum in a 2D gel electrophoresis analysis, and 47 proteins identified in urine in an in-solution analysis. Three proteins, namely, haptoglobin, Rho-related BTB domain containing protein 3, and death-associated protein kinase 3, were selected for further validation by Western blot analysis and results showed that haptoglobin has potential for further development as an additional marker of NVP induced hepatotoxicity.

## 1. Introduction

HIV infection in the absence of therapy is almost invariably fatal with few exceptions, but the introduction of combination antiretroviral therapy (ART) or highly active antiretroviral therapy (HAART) resulted in a dramatic decline in morbidity and mortality that has significantly changed the course of acquired HIV infection [1]. Approximately 35 million people worldwide are living with HIV and an estimated 15% to 30% are coinfected with hepatitis C virus (HCV) [2, 3], although in Thailand the prevalence of HCV coinfection with HIV has been reported as 7.8% [4]. Several studies have reported that HIV induced immunosuppression accelerates the natural history of HCV-related liver disease, and coinfected patients are 3- to 5-fold more likely to develop cirrhosis [5, 6]. Nevirapine (NVP), a nonnucleoside reverse transcriptase inhibitor (NNRTI), is frequently used in the HAART regimen for HIV-infected patients in resource limited settings despite the high risk of hepatotoxicity which occurs in approximately 12–15% of HIV- and HIV/HCV-coinfected patients [7–9].

HIV/HCV-coinfected patients are more likely to develop hepatotoxicity caused by HAART than HIV-monoinfected individuals, and HIV/HCV is associated with a 2–10-fold change of aspartate aminotransferase (AST) and/or alanine aminotransferase (ALT) values above the upper level of normality after starting HAART, compared with HIV infection alone [10]. This is supported by studies that show that HCV is associated with a 2.46 increased relative risk (RR) for liver enzyme elevation (5 × upper limit of normal) [11] and that grades 3-4 liver enzyme elevation was detected in 20.8% of HIV/HCV-coinfected patients who continuously use NVP [12].

NVP hepatotoxicity is believed to be caused by two mechanisms: an early onset reaction characterized by skin reactions and elevated ALT/AST that occurs within the first two to three weeks of treatment [13–15] and a delayed onset that normally starts some 4-5 months after commencement of treatment [15, 16]. While the mechanism of hepatotoxicity remains poorly characterized, NVP metabolites such as 12-OH-NVP and quinone methide have been strongly implicated in the process [17, 18] and evidence for the metabolic activation of NVP through the detection of mercapturates in urine has been previously presented [19].

The early diagnosis of liver toxicity in patients undergoing long term treatment with NVP is critical as the condition can be lethal, and currently this is generally assessed by evaluation of alanine transaminase (ALT) levels [20]. This study aimed to use a proteomic analysis to identify possible biomarkers that are more specific to NVP toxicity than ALT in serum and urine fraction of patients with liver toxicity due to long term nevirapine use.

## 2. Methods

### 2.1. Patients and Samples

#### 2.1.1. Study Design and Participants

The patients included in the study consisted of 18 patients monoinfected with HIV and 13 patients coinfected with HIV and HCV who were being followed up at Maharaj Nakorn Chiang Mai Hospital. Additional five healthy individuals were recruited as controls. All patients received a HAART regimen containing NVP for at least four months. The study was approved by the Research Ethics Committee 2, Faculty of Medicine, Chiang Mai University (RIH-12-985-FB), and the Human Experimentation Committee, Research Institute for Health Sciences (RIHES), Chiang Mai University (4/55). Written informed consent was obtained from all participants.

Blood (10 mL each) and urine samples were obtained on the day of scheduled patient follow-up. Serum samples were prepared and screened for aspartate aminotransferase (AST), alanine transaminase (ALT), hepatitis B surface antigen (HBS-Ag), hepatitis B surface antibody (anti-HBS), anti-hepatitis-C antibodies (anti-HCV), and HIV virus antibody (anti-HIV). All serum and urine samples were stored at −80°C until analysis.

Participants with HIV/HCV with high ALT were eligible to participate if they had received a HAART regimen containing NVP for at least four months, have positive anti-HCV, have positive anti-HIV, were negative for HBS-Ag, were positive/negative for anti-HBS, and have serum ALT more than 1.25-fold of upper normal limit.

Participants with HIV/HCV with normal ALT were eligible to participate if they received a HAART regimen containing NVP for at least four months, have positive anti-HCV, have positive anti-HIV, were negative for HBS-Ag, were positive/negative for anti-HBS, and have serum ALT 0–40 U/L.

Participants with HIV monoinfection with high ALT were eligible to participate if they received a HAART regimen containing NVP for at least four months, have positive anti-HIV, have negative anti-HCV, were negative for HBS-Ag, were positive/negative for anti-HBS, and have serum ALT more than 1.25-fold of upper normal limit.

Participants with HIV monoinfection with normal ALT were eligible to participate if they received a HAART regimen containing NVP for at least four months, have positive anti-HIV, have negative anti-HCV, were negative for HBS-Ag, were positive/negative for anti-HBS, and have serum ALT 0–40 U/L.

The exclusion criteria included positive anti-HBsAg, negative anti-HCV in HIV/HCV coinfection, and less than four months of NVP treatment.

#### 2.1.2. Sample Preparation

Urine samples were thawed at room temperature and centrifuged at 17,000 ×g for 15 min at room temperature to sediment cellular fragments. The supernatants were transferred into new 50 mL tubes and 2 volumes of cold acetone (−20°C) were added to each tube. The tubes were vortexed and subsequently incubated at −20°C overnight after which the samples were centrifuged for 15 minutes at 13,000 ×g. The protein pellets were dissolved in 0.5% SDS and the protein concentration was determined by the Lowry method [21]. Serum samples were diluted 1 : 10 with sterile nano water prior to protein measurement.

Protein samples from serum and urine were subsequently pooled into five groups, namely, HIV/HCV-coinfected patients with high ALT (group 1), HIV/HCV-coinfected patients with normal ALT (group 2), HIV-monoinfected patients with high ALT (group 3), HIV-monoinfected patients with normal ALT (group 4), and healthy individuals (Ctl).

#### 2.1.3. Sub-10 kDa Peptidome Analysis

Approximately 240 *μ*g of pooled proteins from serum or urine was applied directly onto a Nanosep 10 K ultrafiltration device (Pall Life Sciences, Pall Corporation, Port Washington, NY) and the sub-10 kDa fraction removed for protein quantitation by the Lowry assay [21]. A total of 10 *μ*g of protein was transferred into a new tube and the volume adjusted to 10 *μ*L with 10 mM ammonium bicarbonate. The proteins were reduced with 10 mM dithiothreitol in 10 mM ammonium bicarbonate at room temperature for 1 hr and subsequently alkylated with 100 mM iodoacetamide in 10 mM ammonium bicarbonate at room temperature in the dark for 1 hr. Samples were digested with 10 ng trypsin in 50% acetonitrile/10 mM ammonium bicarbonate at room temperature for 3 hrs. The samples were dried under vacuum until the final volume was 10 *μ*L. Then the sample volume was adjusted to 15 *μ*L with 0.1% FA and subjected to LC-MS/MS exactly as described previously [22].

#### 2.1.4. Two-Dimensional Gel Electrophoresis (2-DE) and Liquid Chromatography-Mass Spectrometry Analysis

Approximately 60 *μ*L of each of the pooled serum samples was taken and processed to deplete albumin using the ProteoExtract Albumin removal kit (Merck KGaA, Darmstadt, Germany) according to the manufacturers' recommendations and protein concentrations were determined as described previously. Two-dimensional gel electrophoresis and LC-MS/MS analysis were performed essentially as described previously [23]. Briefly, 200 *μ*g of each pooled albumin depleted fraction was mixed with 340 *μ*L of rehydration buffer (8 M urea, 4% CHAPS, 0.001% bromophenol blue, and 3 mM dithiothreitol) containing 1% 3–10 NL IPG buffer. Samples were loaded onto 18 cm IPG strips with pH range of 3–10 NL of an isoelectric focusing system (EttanIPGphoreIII). Strips were rehydrated (20°C, 16 h) and isoelectric focused (500 volts for 500 volt-h, 1,000 volts for 800 volt-h, and 10,000 volts to reach 36,000 volt-h). The maximum current was maintained at 75 *μ*A per strip. Strips were subsequently equilibrated twice (15 min each) in equilibration buffer (50 mM Tris pH 8.8, 6 M urea, 30% glycerol, 2% SDS, and 0.03% bromophenol blue) supplemented with 65 mM DTT and 135 mM iodoacetamide. The strips were subjected to the second dimensional separation (Ettan DALTsix) using a SDS-polyacrylamide gel (12.5%) run under an applied voltage of 10 watts per gel at 20°C until the bromophenol blue dye front reached 0.5 cm from the bottom of the gel. The gels were subsequently stained with colloidal Coomassie blue. Spots identified as differentially regulated were removed from the blot and subjected to “in-gel” tryptic digestion as described previously [23]. Peptides were identified by LC-MS/MS using a Dionex Ultimate 3000 (Thermo Scientific) in combination with an electrospray ionization (ESI)/quadrupole ion trap mass spectrometer (amaZon SL, Bruker Daltonik, Germany). The LC separation was performed on a reverse phase column (Hypersil GoLD 50 × 0.5 mm, 5 *μ*m C18), protected by a guard column, and eluted at a flow rate of 100 *μ*L/min under gradient conditions of 5–80%B over 50 min. The mobile phase A consisted of water/formic acid (99.9 : 0.1, v/v), and mobile phase B consists of acetonitrile (100, v). Mass spectral data from 150 to 1500* m/z* was collected in the positive ionization mode. The MS/MS spectrometry data were searched against the NCBInr database using the MASCOT search engine as described previously [22, 23].

#### 2.1.5. Protein Preparation and Western Blotting

Sera were individually albumin depleted as above and concentrated using a Vivaspin 2 ultrafiltration column with a 10 kDa molecular weight cut-off (GE Healthcare, Buckinghamshire, UK) according to the manufacturer's recommendations. Protein concentration then was determined as before and samples were stored at −80°C until required. SDS-PAGE and Western blot analysis were employed to determine the enrichment of serum. Approximately 50 *μ*g proteins were subjected to electrophoresis on 10% SDS-polyacrylamide gels and transferred to PVDF solid matrix support (Amersham Hybond-P, GE Healthcare) and analyzed as previously described [23]. The primary antibodies used were anti-RhoBTB3 (Sigma-Aldrich Co., St. Louis, MO), anti-ZIP kinase antibody (DAPK3) (Abcam plc, Cambridge, UK), anti-haptoglobin (Abcam plc), anti-transferrin (Abcam plc), and anti-GAPDH (Cell Signaling Technology, Inc., Danvers, MA). The secondary antibodies used were a goat anti-rabbit IgG-HRP (Santa Cruz Biotechnology Inc., Santa Cruz, CA) and a goat anti-mouse IgG-peroxidase (Sigma-Aldrich, Milwaukee, WI). Blots were visualized using Luminata Forte Western HRP Substrate (Merck KGaA, Darmstadt, Germany) detection system, according to the manufacturer's instructions. Antibodies against transferrin and/or GAPDH were employed as internal controls. Image J software was employed to determine optical density values of bands for relative comparisons. The experiment was performed in triplicate.

### 2.2. Statistical Analysis

The data were expressed as mean ± SD. To compare clinical parameters and the expression level of proteins among patients and control, statistical significance of bands intensity was determined using one-way ANOVA with Bonferroni's multiple comparison test using Prism 5. *P* values less than 0.05 were considered statistically significant.

## 3. Results

### 3.1. Study Participants and Clinical Parameters of Participants

**Figure 1:**
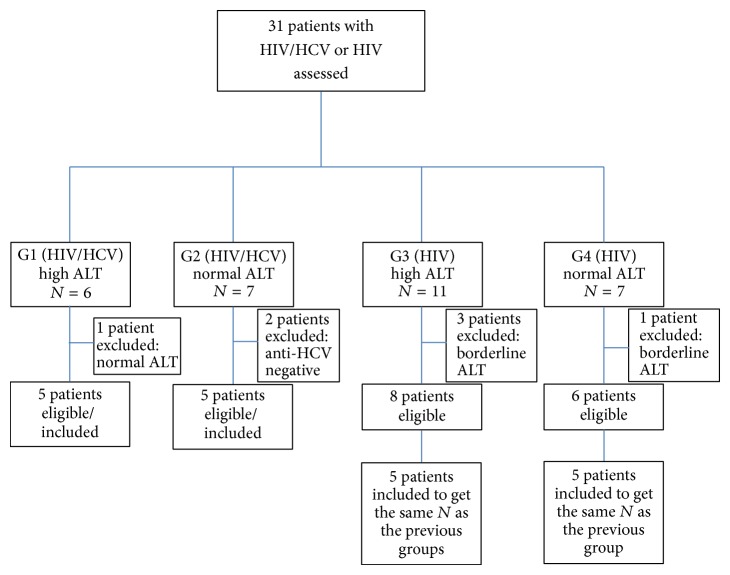
Schematic of patient inclusion and exclusion.

**Figure 2:**
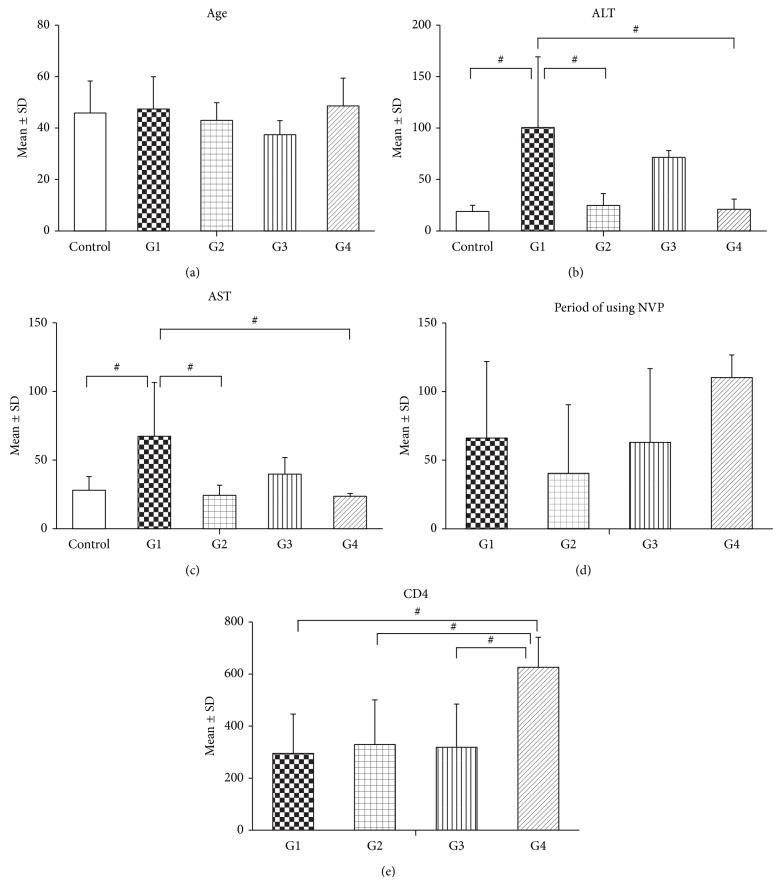
Means ± SD of clinical parameters. The information was obtained from 5 HIV/HCV-coinfected patients with high ALT (G1), 5 HIV/HCV-coinfected patients with normal ALT (G2), 5 HIV-monoinfected patients with high ALT (G3), 5 HIV-monoinfected patients with normal ALT (G4), and 5 healthy controls (Ctl). ALT: alanine transaminase; AST: aspartate aminotransferase. All values are reported as means ± SD. One-way ANOVA with Bonferroni's multiple comparison test was performed; statistical significance is shown by # for *P* < 0.05.

Between June 22, 2012, and August 1, 2012, a total of 31 HIV/HCV and HIV adults were screened for enrollment in the study (Figure 1). A total of 11 patients (35.5%) were excluded due to the presence of normal ALT (1), negative anti-HCV (2), borderline ALT level (4), and a further 4 were not included in the analysis to give equal *N* values in the four patient groups. In addition, five healthy controls were recruited. All patients had had normal baseline ALT levels before commencement of NVP therapy. Based upon levels of ALT at the time of sample collection, patients were divided into four groups, HIV/HCV-coinfected patients with high ALT (group 1), HIV/HCV-coinfected patients with normal ALT (group 2), HIV-monoinfected patients with high ALT (group 3), HIV-monoinfected patients with normal ALT (group 4), and healthy controls represented a fifth group (Ctl).  Figure 2 shows the characteristics of each group for age, ALT, AST, period of using NVP, and CD4+ cell count. No significant difference was observed between all groups for age distribution, and similarly no difference was observed in the patient groups for the period of NVP usage. Group 1 had significantly higher ALT and AST levels than the control group as well as groups 2 and 4. Group 4 had a significantly higher mean CD4+ cell count than groups 1, 2, and 3.

### 3.2. Urine Proteomic Analysis

Sub-10 kDa protein fractions prepared from pooled urine samples of the five groups were subjected to in-solution tryptic digestion followed by LC-MS/MS analysis. A total of 318 proteins were identified, of which 47 were differentially expressed between groups (Table 1). The majority of differentially expressed proteins were identified as being involved in transcription (24%), signal transduction (13%), transport (11%), and immune responses (11%). The remaining proteins were associated with a range of diverse functions. Some 32% of the identified proteins were cytoplasmic, with 21% being membrane proteins and a further 24% were identified as nuclear proteins.

Of the 47 differentially expressed proteins, 33 were found in all groups while 14 proteins were identified only in some groups. Of the 14 proteins found in only some groups, seven (Sacsin, carboxy-terminal domain RNA polymerase II polypeptide A small phosphatase 3, KIAA0591 protein, metabotropic glutamate receptor 5 isoform b precursor, UBA3, vacuolar protein sorting-associated protein 45, and E3 ubiquitin-protein ligase HECTD1) were only found in patients groups and were absent from controls, although the level of the 7 proteins was similar between the patient groups (data not shown). The remaining 7 proteins showed no discernible trend for association with NVP treatment and liver damage.

### 3.3. Serum Proteomic Analysis: In-Solution

LC-MS/MS analysis of the sub-10 kDa fraction of pooled serum samples revealed a total of 251 identified proteins, of which 34 were found to be differentially expressed. Details of the 34 proteins and their biological function are presented in Table 2. The majority of differentially expressed proteins were associated with transport (14%), cell development (12%), apoptosis (9%), cell adhesion (9%), lipid metabolism (9%), signal transduction (9%), and transcription (9%). The identified proteins were primarily identified as cytoplasmic (47%) and membrane proteins (20%).

Twenty-seven of the differentially expressed proteins were found in all groups. Seven proteins (Rho-related BTB domain containing protein 3 (RhoBTB3), zinc finger and BTB domain containing protein 44 (ZTBT44), obscurin isoform B, BAI 1, RNA exonuclease 1 homolog, death-associated protein kinase 3 (DAPK3), and chondroitin sulfate proteoglycan 4 protein (CSPG4)) showed restricted expression in 1 to 3 groups.

The peptide intensity analysis showed (Table 3) that RhoBTB3 was detected only in the high ALT groups from both HIV/HCV coinfection and HIV monoinfection and was not detected in the normal ALT groups or in normal control samples. The peptide intensity in the HIV/HCV-coinfected group (group 1) was approximately double that found in the monoinfected group (group 3). DAPK3 and CSPG4 were both highly expressed in control and greatly reduced or absent in the patient groups. The remaining peptide intensity distributions were largely uninformative, and so RhoBTB3 and DAPK3 were selected for validation by Western blot analysis.

### 3.4. Serum Proteomic Analysis: In-Gel

Albumin depleted pooled serum samples from the five groups were subjected to 2D gel electrophoresis and the gels were stained with Coomassie blue (data not shown). Two spots were identified, one of which was only expressed in group 1 while the second was observed to be significantly downregulated in group 1 as compared to the other groups, including control. The two spots were excised from the gels, subjected to tryptic digestion, and the resultant peptides analyzed by LC-MS/MS. Resultant data was searched against the SwissProt database for protein identification using the Mascot software (http://www.matrixscience.com/). The proteins were identified as transthyretin and haptoglobin, respectively.

## 4. Validation of Serum Biomarkers Using Western Blotting

**Figure 3:**
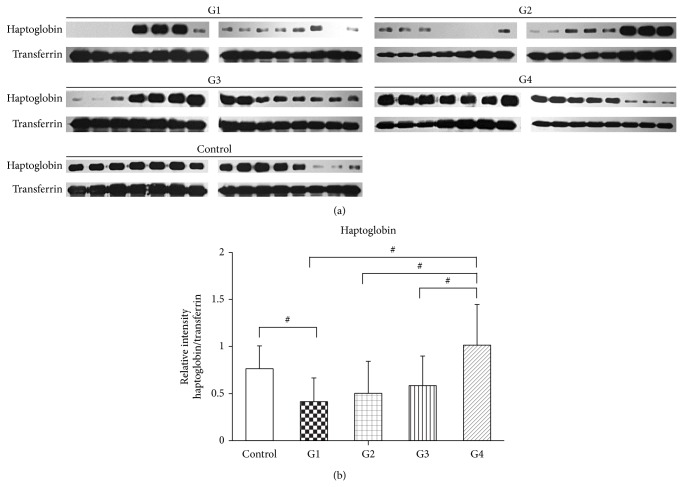
Western blot analysis and quantitation of haptoglobin expression in serum of normal controls and HIV patients undergoing long term NVP treatment. (a) Serum samples were individually depleted for albumin and proteins run as triplicate replicates before transfer to solid matrix support and western analysis to determine haptoglobin expression. Filters were reprobed after stripping with an antibody against transferrin to provide a loading control. G1 (group 1; HIV/HCV-coinfected patients with high ALT), G2 (group 2; HIV/HCV-coinfected patients with normal ALT), G3 (group 3; HIV-monoinfected patients with high ALT), G4 (group 4; HIV-monoinfected patients with normal ALT), and control (healthy controls). (b) Quantitation was determined by image analysis and is expressed as relative intensity (haptoglobin/transferrin). Data is plotted as means ± SD. Statistical significance was determined using one-way ANOVA with Bonferroni's multiple comparison test. # designates *P* < 0.05.

From the results of the in-solution and in-gel proteomic analyses of serum, three proteins (DAPK3, RhoBTB3, and haptoglobin) were selected for confirmation by Western blotting analysis. Each of the 25 serum samples was individually albumin depleted and proteins were separated by electrophoresis before transfer to solid matrix support and Western blot analysis. Levels of GAPDH or transferrin were additionally determined as an internal control. Results showed that haptoglobin (Figure 3) was significantly reduced in group 1 (HIV/HCV-coinfected patients with high ALT). Levels of serum haptoglobin in group 4 (HIV-monoinfected patients with normal ALT) were similar to levels in the control group and significantly above the levels seen in the three remaining patients groups (HIV/HCV-coinfected patients with high ALT, HIV/HCV-coinfected patients with normal ALT, and HIV-monoinfected patients with high ALT). The largest difference in haptoglobin levels was seen between group 1 (HIV/HCV-coinfected patients with high ALT) and group 4 (HIV-monoinfected patients with normal ALT). While some slight differences were noted between the groups for both RhoBTB3 and DAPK3, the differences did not reach statistical significance.

## 5. Discussion

A highly active antiretroviral treatment (HAART) regimen containing NVP appears to be associated with a higher risk for increased liver damage in HIV-1-infected patients who are coinfected with HCV as compared to monoinfected HIV patients, and as such specific biomarkers that can be used in conjunction with ALT would be useful as an adjunct marker to ALT alone. Towards this end, this study undertook a proteomic analysis of serum and urine from HIV-monoinfected and HIV/HCV-coinfected patients undergoing long term NVP treatment in parallel with control samples.

The patient samples were analyzed in four groups, namely, HIV/HCV coinfection with high ALT (group 1), HIV/HCV coinfection with normal ALT (group 2), HIV monoinfection with high ALT (group 3), and HIV monoinfection with normal ALT (group 4). The patients in the high ALT cohorts all had grade 1 liver elevation except two patients in group 1 who had grade 2 elevation. No higher ALT grade patients were available as their regimes are normally altered to prevent further liver damage. As such, this study was somewhat limited in the range of liver hepatotoxicity studied and the study should be further cautiously interpreted in light of the small number of patients in each group.

A total of 83 proteins were identified through the three arms of the study (in-solution analysis of serum and urine and in-gel analysis of serum), although no protein was identified consistently in all three arms of the study, presumably as a result of differing sample and methodological approach. The two proteins selected for further validation from those identified from the in-solution proteomic analysis (RhoBTB3 and DAPK3) showed only slight and nonsignificant differences between the four patient groups. The third protein, haptoglobin, identified from the in-gel 2D electrophoresis assay showed slight but not statistically significant elevation in monoinfected HIV patients with normal ALT and statistically significantly reduced levels compared to normal controls in HIV/HCV-coinfected patients with high ALT. Levels of haptoglobin were slightly reduced in HIV/HCV-coinfected patients with high ALT (group 1) as compared to HIV/HCV-coinfected patients with normal ALT (group 2) although this was not statistically significant. However, as noted previously, the absence of grades 3 and 4 ALT levels probably reduces the discriminating ability of the analysis.

The plasma protein, haptoglobin, is synthesized by hepatocytes and functions to noncovalently bind oxidized hemoglobin generated by hemolysis [24]. The hemoglobin-haptoglobin complex is taken up by the reticuloendothelial system both to scavenge iron for recycling and to prevent adverse oxidative effects [24]. A significant decrease in haptoglobin levels during ART has been previously reported [25], and the decrease was unrelated to hemophilia or HCV status, and was not associated with the presence of increased markers of hemolysis. In our study, we observed a significant decrease in haptoglobin levels in patients with HIV/HCV coinfection and high ALT and nonsignificant reductions in patients with HIV/HCV coinfection and normal ALT and in monoinfected HIV patients with normal and high ALT. A significant difference was seen between levels of haptoglobin in monoinfected HIV patients with high and normal ALT, and while it did not reach statistical significance, the levels of haptoglobin were reduced in HIV/HCV-coinfected patients with high ALT as compared to HIV/HCV-coinfected patients with normal ALT. These results suggest that reduced levels of haptoglobin may reflect increased liver damage. In this regard, haptoglobin has previously been reported as a serum marker of fibrosis in chronic hepatitis C patients [26], where it is believed that liver fibrosis leads to increased expression of hepatocyte growth factor promoting a subsequent decrease in haptoglobin levels.

Studies have suggested that serum haptoglobin predicts liver fibrosis in hepatitis C patients where it was found that haptoglobin was one of the top candidate proteins for discriminating grade 0 from grades 1–4 liver fibrosis as well as discriminating grade 3 from grade 4 [27], while another study reported that haptoglobin serum levels were negatively correlated to scores of fibrosis (*P* < 0.001) and suggested that determination of haptoglobin serum level may be useful in the follow-up of patients with chronic hepatitis C [28].

Although the patients in this project were not yet in fibrotic stages, a correlation between liver status and haptoglobin was observed in group 1 in comparison with group 2 HCV patients. The patients in group 1 have more severe grade 2 liver injury as compared to the patients in group 2 that contained only grade 1 liver injury and showed lower haptoglobin levels. However, this was not statistically significant, possibly since only two out of five patients in group 1 were grade 2 for liver injury. Interestingly, patient group 3 (monoinfected with HIV with high ALT) had relatively low level of haptoglobin compared to group 4 who had normal ALT suggesting that haptoglobin may be an earlier marker for liver injury caused by NVP in HIV patients. Haptoglobin is one of five parameters (alpha2-macroglobulin, haptoglobin, apolipoprotein A1, gamma glutamyl transpeptidase, and bilirubin) used to evaluate liver fibrosis stage in the Fibrotest [29] and an inverse relationship between haptoglobin and bilirubin levels have been reported in hepatitis patients although the relationship was dependent upon the type of liver disease [30].

Overall, this study identified a number of possible candidate biomarkers of NVP induced liver toxicity, one of which (haptoglobin) was successfully validated. Two potential biomarkers failed Western blot validation (RhoBTB3 and DAPK3) but remain of interest for possible future validation in larger cohorts of patients.

## Figures and Tables

**Table 1:** List of proteins detected as differentially regulated in urine of patients undergoing long term NVP treatment and normal controls.

Protein name	Database ID number	Protein ID score	Function	Localization	Mass (Da)	Peptide sequence
ADAMTS12	gi∣124297123	3.21	Protein metabolism	Unknown	1036.6585	KCNQQACK
Sacsin	gi∣327200585	2.49	Immune response	Membrane	911.5248	FTSEVAXR
NADPH oxidase	gi∣34810991	2.66	Immune response	Cytoplasm	722.5438	YTVVXK
Ubiquitin-conjugating enzyme E2	gi∣269914374	2.24	Ubiquitin metabolism	Cytoplasm	849.2900	AGSMALKR
Carboxy-terminal domain RNA polymerase II polypeptide A small phosphatase 3	gi∣2289786	12.59	Transcription	Nucleus	672.2483	LPGAGEK
dynein	gi∣119627741	4.40	Single	Cytoplasm	733.7764	LFAQVR
Dystroglycan	gi∣294997282	9.68	Cytoskeleton	Cytoplasm	679.6234	KPPLPK
E3 ubiquitin-protein ligase HECTD1	gi∣118498337	22.38	Ubiquitin metabolism	Cytoplasm	870.7503	KASLALIR
FL100154	gi∣18676514	6.78	Others	Unknown	1532.2699	PEMTGVTVSGLTPAR
hCG1783738	gi∣119606481	1.35	Others	Unknown	756.2752	KPALGNR
hCG2042782	gi∣119574283	5.61	Others	Unknown	972.8246	VAMTVIKPV
hCG2045758	gi∣119626212	5.75	Others	Unknown	867.1841	RVASAYLS
Protein HEG homolog 1	gi∣33871486	8.09	Cell development	Membrane	850.1389	LFDLADR
hydroxyacylglutathione hydrolase	gi∣119606016	3.62	Carbohydrate metabolism	Mitochondria	782.3398	VYGGDDR
Hypothetical protein	gi∣60219224	7.34	Others	Unknown	1568.0209	DMQMGPGLFLRMR
IgA nephropathy-related protein 1	gi∣307133516	5.16	Single	Membrane	795.7409	GPVSPPNK
Immunoglobulin heavy chain VHDJ region	gi∣21669823	1.48	Immune response	Cytoplasm	773.9408	RGGVGTTK
Immunoglobulin kappa light chain	gi∣62860960	3.36	Immune response	Cytoplasm	920.3153	TVAAPSVFK
Keratin	gi∣32567786	17.72	Cell development	Cytoplasm	701.4250	MDLHGK
KIAA0591	gi∣29421178	4.69	Apoptosis	Mitochondria	833.9171	SYTMMGK
KIAA0895	gi∣4240279	5.22	Others	Unknown	835.4904	DIGRFVK
Patched domain containing protein	gi∣326205357	3.36	Transport	Membrane	674.6955	LSKNGR
KIAA1409	gi∣119601937	26.30	Response to stress	Membrane	856.7432	IAVSAIQR
KIAA2010	gi∣24899184	1.10	Protein metabolism	Cytoplasm	902.6365	EDTLPLSK
Metabotropic glutamate receptor 5	gi∣4504143	7.70	Single	Nucleus	1406.2405	YEIMNFKEMGK
MHC class 1 antigen	gi∣123278215	7.54	Immune response	Membrane	688.0275	QAPTDR
Myosin light chain kinase 2	gi∣14043538	3.01	Transport	Cytoplasm	1434.2871	DFVSNLIVKDQR
Peroxisome proliferator-activated receptor alpha	gi∣1514595	1.01	Transcription	Nucleus	758.7757	FDFAMK
Progesterone-induced-blocking factor 1	gi∣3925685	6.64	Transcription	Cytoplasm	986.7480	QNSLIXKR
Pleckstrin homology domain containing family A member 6	gi∣37595548	2.93	Lipid metabolism	Cytoplasm	757.5746	SVPQAVR
Poly(ADP-ribose) polymerase 1	gi∣119590191	7.36	Transcription	Nucleus	1475.5247	LQGHVPQTSLQLR
Suppressor of cytokine signaling 5	gi∣23273934	3.30	Single	Cytoplasm	783.9406	RDSYSR
REST corepressor	gi∣344925845	5.18	Transcription	Nucleus	673.1253	GPEVSGK
REST corepressor 3	gi∣21595082	7.97	Transcription	Nucleus	714.2440	GPELLGK
Uncharacterized protein FLJ37004	gi∣74729710	3.79	Others	Unknown	1519.9062	ATAAAARGSEVSGEGGK
Serine/threonine protein kinase RIO3	gi∣119621558	2.00	Transport	Membrane	1263.2434	ENVGDGIGMDLK
Serine/threonine protein kinase PLK4	gi∣2125814	2.52	Single	Cytoplasm	866.4060	DRASFNR
SEC14-like 4	gi∣119580300	7.04	Transport	Membrane	819.6610	SSATLWR
SH2 domain containing protein 3C	gi∣4704739	3.24	Single	Membrane	658.6454	TAVGRR
Sorting nexin-3	gi∣3126979	2.35	Transport	Membrane	1017.4337	AETVADTRR
NEDD8-activating enzyme E1 catalytic subunit	gi∣3342564	1.45	Transcription	Nucleus	744.0996	LTQGVVK
Uncharacterized protein	gi∣254692913	2.93	Others	Unknown	1000.3987	RLLNELDK
Ribosomal RNA upstream binding transcription factor	gi∣1916615	2.03	Transcription	Nucleus	780.6412	LRGPNPK
Vacuolar protein sorting-associated protein 45	gi∣18105063	5.13	Transcription	Nucleus	838.4481	KMSGTVSK
WD repeat-containing protein 46	gi∣256773176	7.41	Transcription	Nucleus	696.2765	KPQVPK
Coiled-coil domain containing protein 105	gi∣226492892	2.14	Transcription	Nucleus	540.5465	APTPR
17-*beta*-Hydroxysteroid dehydrogenase 14	gi∣59889578	7.76	Cell division	Cytoplasm	506.0660	ATGTR

**Table 2:** List of proteins detected as differentially regulated in serum of patients undergoing long term NVP treatment and normal controls.

Protein name	Database ID number	Protein ID score	Function	Localization	Mass (Da)	Peptide sequence
BAI 1	gi∣2653432	10.04	Cell adhesion	Membrane	857.1950	NATGLILR
BCL2/adenovirus E1B 19 kDa interacting protein	gi∣119573869	2.14	Apoptosis	Nucleus	878.1002	VFRMGPR
Catenin alpha-1	gi∣21411495	6.22	Cell adhesion	Cytoplasm	1492.7295	EYAQVFREHANK
Death-associated protein kinase 3	gi∣4557511	1.79	Apoptosis	Cytoplasm	617.4699	QKGTGK
Zinc finger protein 174	gi∣159163639	7.90	Transcription	Nucleus	742.0348	CLMSSK
Collagen type X11 alpha-1	gi∣1846005	15.77	Cell adhesion	Unknown	1554.9274	GGNTMTGDAIDYLVK
Corin	gi∣4884872	2.91	Protein metabolism	Membrane	1474.3988	WVLTVAHCFEGR
Chondroitin sulfate proteoglycan 4	gi∣124504610	3.64	Carbohydrate metabolism	Membrane	1551.0338	TGKHDVQVLTAKPR
FYVE and coiled domain containing protein 1	gi∣13276231	4.84	Transport	Cytoplasm	874.5961	AALDDQDK
hCG1811779	gi∣119578948	2.12	Others	Unknown	701.1700	LTGPAGGK
hCG2018146	gi∣119595992	1.36	Others	Unknown	815.1358	CLLRPR
hCG2033821	gi∣119618839	2.35	Others	Unknown	707.6396	TATEMR
Obscurin isoform B	gi∣89143259	1.77	Apoptosis	Cytoplasm	1002.3102	KKPGLASFR
Eukaryotic translation initiation factor 4B	gi∣48146033	3.18	Translation	Cytoplasm	828.0649	EPSNPER
Coiled-coil domain containing 146	gi∣23271918	4.84	Cytoskeleton	Cytoplasm	955.2563	ELVVNLLR
Sodium dependent glucose transporter 1	gi∣154937330	5.29	Transport	Membrane	982.5936	HLPETRTK
Microtubule associated protein 1a	gi∣1790878	2.47	Cytoskeleton	Cytoplasm	849.2162	DKDLEXK
Myosin VI	gi∣9280816	1.43	Cell development	Cytoplasm	519.0438	AAAGTK
Peroxisomal *trans*-2-enoyl-coA reductase	gi∣19923817	7.82	Lipid metabolism	Cytoplasm	846.0755	GAGDLSVVK
Phospholipase B domain containing protein 1	gi∣15029600	3.14	Lipid metabolism	Cytoplasm	927.4162	MMADSGKR
Calmodulin-regulated spectrin-associated protein 2	gi∣44955929	20.38	Cytoskeleton	Cytoplasm	701.6011	AGSLILK
C-type lectin domain family 4 member K	gi∣305410858	12.79	Immune response	Cytoplasm	702.7117	ASALNTK
Zinc finger and BTB domain containing protein 44	gi∣30047807	20.92	Transcription	Nucleus	905.7966	VQDKIFR
RNA exonuclease 1 homolog	gi∣145199237	10.68	DNA replication	Nucleus	821.9409	ASSRDER
Rhesus blood group D antigen	gi∣28629452	6.69	Transport	Membrane	691.7388	VVITLF
Rho-related BTB domain containing 3	gi∣27371219	5.84	Singal	Membrane	1897.7174	CEVMAAMFNGNYMEAK
Solute carrier family 4, sodium bicarbonate cotransporter, member 5	gi∣119620095	24.26	Transport	Membrane	856.6955	SPSSLLPR
Tyrosine-protein phosphate non receptor type 23	gi∣24308073	3.39	Single	Cytoplasm	1008.7161	HVEQVLQR
Zinc finger protein 556	gi∣13376460	4.83	Transcription	Nucleus	710.6905	CGKCGK
Dynactin 1	gi∣4139121	3.52	Transport	Cytoplasm	515.6265	APTAR
Casein kinase I isoform gamma-1	gi∣98986450	7.18	Single	Cytoplasm	685.1477	HIPYR
hCG2045456	gi∣119605796	2.79	Others	Unknown	671.2872	EFNNF
Group IIF secretory phospholipase A2	gi∣145553989	3.89	Lipid metabolism	Cytoplasm	462.4213	ADGAK
hCG2038093	gi∣119602010	3.27	Others	Unknown	613.7015	HVSRD

**Table 3:** Peptide intensities of 7 proteins showing differential expression of serum proteins between groups.

Protein	Group 1	Group 2	Group 3	Group 4	Control
RhoBTB3	20.03	0	9.20	0	0
ZTBT44	0	5.49	8.48	16.72	0
Obscurin isoform B	17.40	5.76	3.58	0	28.70
BAI 1	17.30	4.69	1.60	0	28.86
RNA exonuclease 1 homolog	19.05	0	9.10	16.11	29.70
DAPK3	0	7.61	10.61	14.59	30.44
CSPG4	0	6.15	0	12.80	30.66

Group 1: HIV/HCV-coinfected patients with high ALT; Group 2: HIV/HCV-coinfected patients with normal ALT; Group 3: HIV-monoinfected patients with high ALT; Group 4: HIV-monoinfected patients with normal ALT; Control: healthy controls. Seven proteins RhoBTB3: Rho-related BTB domain containing protein 3; ZTBT44: zinc finger and BTB domain containing protein 44; DAPK3: death-associated protein kinase 3; CSPG4: chondroitin sulfate proteoglycan 4. Peptide intensity values are shown to 2 decimal places.
